# Suspected Biphasic Anaphylaxis Following the First Known Dose of Tirzepatide: A Case Report and Brief Review of Reported Hypersensitivity Reactions

**DOI:** 10.7759/cureus.113910

**Published:** 2026-08-03

**Authors:** Abdulmuhsun H Al-Rashid, Fatima Ali

**Affiliations:** 1 General Practice, Mubarak Al-Kabeer Hospital, Jabriya, KWT; 2 Immunology, Faculty of Medicine, Kuwait University, Jabriya, KWT

**Keywords:** adverse reactions, anaphylaxis, clinical case report, hypersensitivity, tirzepatide

## Abstract

Tirzepatide is a dual glucose-dependent insulinotropic polypeptide and glucagon-like peptide-1 receptor agonist increasingly used for type 2 diabetes mellitus and chronic weight management. Although generally well tolerated, serious systemic hypersensitivity reactions may occur. We report a 37-year-old woman who developed anaphylaxis shortly after administering her first known subcutaneous dose of tirzepatide. She presented with facial and periorbital angioedema, nasal congestion, generalized urticaria, bronchospasm, hypoxemia, and hypotension. She was treated with intravenous fluids, nebulized bronchodilators, and two doses of intramuscular epinephrine, resulting in complete clinical resolution. Approximately one hour later, she developed recurrent generalized urticaria, facial angioedema, and chest tightness without further exposure to the suspected trigger. Although no objective respiratory or hemodynamic compromise was documented during the recurrent episode, the recurrence was considered suggestive of a possible biphasic reaction. Her symptoms resolved following treatment with intravenous hydrocortisone and chlorphenamine. Acute and baseline serum tryptase levels were unavailable, and validated allergy testing for tirzepatide was not performed; therefore, the underlying immunological mechanism could not be established. This case highlights the importance of early recognition and prompt treatment of tirzepatide-associated anaphylaxis, as well as continued pharmacovigilance and detailed reporting of severe hypersensitivity reactions.

## Introduction

Tirzepatide is a dual agonist of the glucose-dependent insulinotropic polypeptide (GIP) and glucagon-like peptide-1 (GLP-1) receptors used in the treatment of type 2 diabetes mellitus and chronic weight management [1,2]. Structurally, it is a synthetic 39-amino-acid peptide based on the native GIP sequence, modified with a C20 fatty diacid moiety to prolong its half-life. Its mechanism of action involves dual activation of GIP and GLP-1 receptors, enhancing glucose-dependent insulin secretion, suppressing glucagon secretion, delaying gastric emptying, and centrally reducing appetite [1,2]. With the rapid clinical adoption of tirzepatide, its safety profile has attracted growing attention [3].

Anaphylaxis is a severe, life-threatening systemic hypersensitivity reaction characterized by rapid onset and multi-organ system involvement [4]. It is most commonly mediated by IgE-dependent mast cell and basophil degranulation, though non-IgE-mediated (immunological or non-immunological) pathways also occur [4,5]. Upon activation, these cells rapidly release preformed vasoactive mediators - primarily histamine, tryptase, chymase, and carboxypeptidase A3 - alongside newly synthesized leukotrienes and prostaglandins [4]. These mediators drive the classic clinical manifestations of anaphylaxis across multiple organ systems, including cutaneous manifestations (pruritus, flushing, urticaria, and angioedema), respiratory manifestations (rhinitis, laryngeal edema, and bronchospasm (wheezing and chest tightness)), cardiovascular manifestations (vasodilation, capillary leak, tachycardia, and distributive hypotension), and gastrointestinal manifestations (nausea, vomiting, and abdominal cramping).

The global incidence of anaphylaxis is estimated at 50-112 episodes per 100,000 person-years, with drug-induced reactions representing a significant proportion of adult cases [4,6]. While mortality is estimated at less than one per million population annually, unpredictable drug-induced reactions demand high clinical vigilance [6,7]. The most common triggers of drug-induced anaphylaxis include beta-lactam antibiotics, nonsteroidal anti-inflammatory drugs (NSAIDs), neuromuscular blocking agents, and radiocontrast media [4,5]. Delayed drug hypersensitivity reactions, which typically manifest days to weeks after exposure as T-cell-mediated cutaneous eruptions, such as drug reaction with eosinophilia and systemic symptoms (DRESS) or Stevens-Johnson syndrome, should be distinguished from immediate hypersensitivity reactions.

Hypersensitivity reactions have been reported with tirzepatide in clinical trials and post-marketing surveillance and are predominantly non-serious; however, serious reactions, including anaphylaxis and angioedema, have also been reported. The frequency of tirzepatide-associated anaphylaxis remains uncertain [1-3,8]. A recent retrospective observational analysis identified 43 published cases of adverse drug reactions associated with tirzepatide across gastrointestinal, endocrine, hepatic, and dermatologic systems [3,8]. Very few case reports of tirzepatide-associated systemic hypersensitivity or anaphylaxis have been published [9,10].

## Case presentation

A 37-year-old Egyptian woman with a history of migraine and temporomandibular joint disorder presented to the emergency department with a rapid-onset clinical constellation of generalized urticaria, angioedema, and respiratory distress. Approximately 10 minutes prior to symptom onset, she had self-administered her first subcutaneous dose of tirzepatide 2.5 mg into the right side of her abdomen, which had been prescribed by her physician for weight management. The injection pen was new and intact and had been stored correctly. She denied previous use of tirzepatide or any other GLP-1 receptor agonist. She denied potential anaphylaxis cofactors, including recent exercise, acute infection, and alcohol consumption. She also denied any new food or medication exposure.

The reaction began with sudden-onset facial and periorbital angioedema, palpitations, nasal congestion, and severe wheezing accompanied by chest tightness. Symptoms rapidly progressed to generalized, pruritic urticarial wheals across her face, trunk, and extremities, accompanied by profound dizziness, prompting emergency medical evaluation.

**Figure 1:**
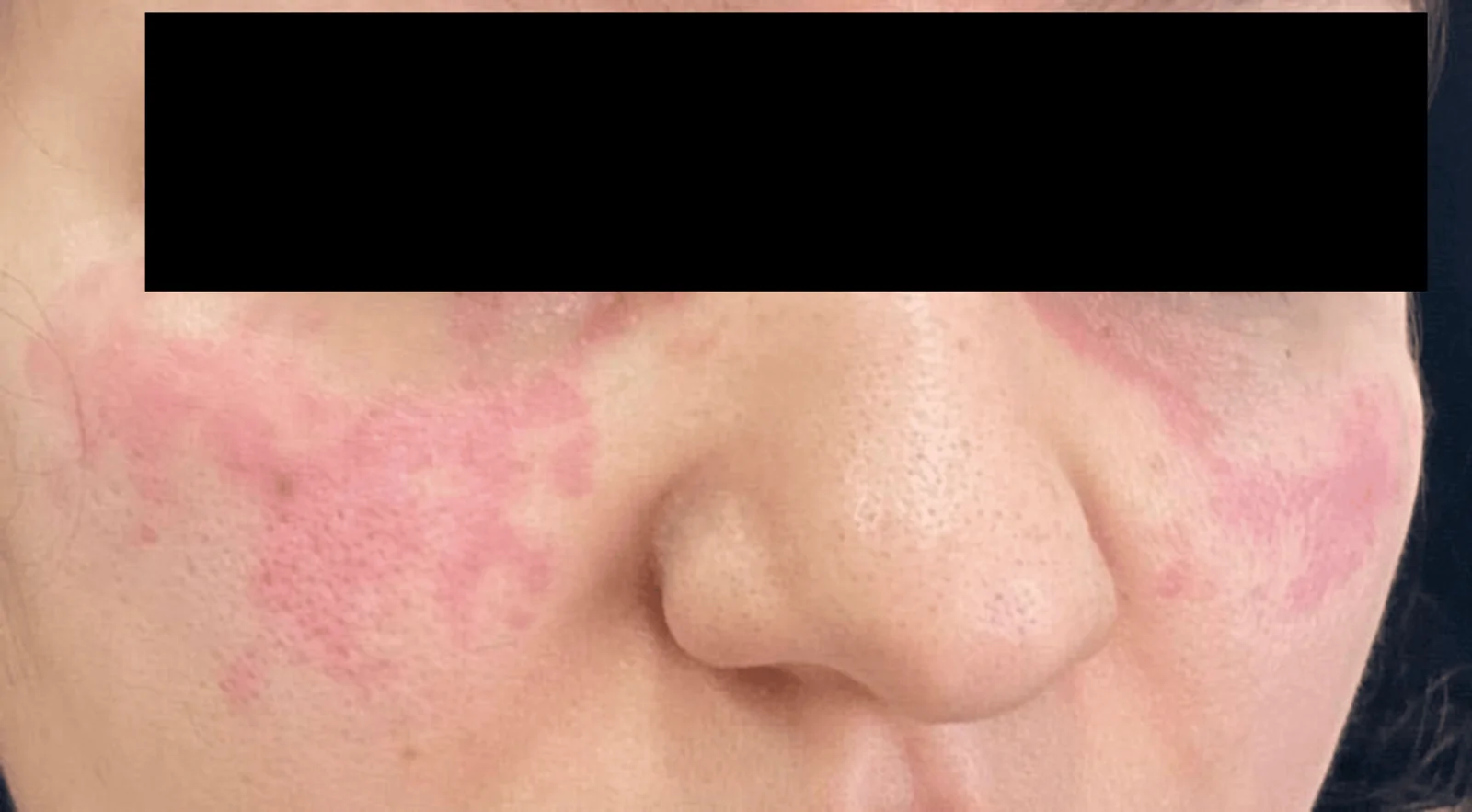
Facial urticaria and erythema observed following administration of the first dose of tirzepatide.

Upon arrival at the emergency department, approximately 30 minutes after symptom onset, the patient demonstrated severe hemodynamic compromise and respiratory distress. Vital signs revealed a blood pressure (BP) of 80/50 mmHg, a heart rate (HR) of 123 beats/min, a respiratory rate of 24 breaths/min, and an oxygen saturation of 92% on room air. Physical examination demonstrated prominent facial angioedema, widespread urticarial wheals (Figure 1), and diffuse bilateral expiratory wheezing on pulmonary auscultation.

The patient immediately received a rapid intravenous infusion of 1 L of 0.9% sodium chloride and an initial dose of intramuscular epinephrine 0.3 mg, using a 1 mg/mL (1:1000) solution, administered into the left mid-anterolateral thigh. She also received nebulized albuterol 2.5 mg, ipratropium 0.5 mg, and budesonide 1 mg. Because hypotension and respiratory compromise persisted after five minutes, a second intramuscular dose of epinephrine 0.3 mg was administered at the same site. This resulted in significant clinical improvement, with stabilization of her BP, resolution of bronchospasm, and gradual improvement in the urticaria and angioedema. Intravenous fluid administration was continued. Within 10 minutes of the second epinephrine dose, her respiratory symptoms and cutaneous manifestations had resolved. Her BP was 110/78 mmHg, her HR was 90 beats per minute, and her oxygen saturation was 97% while receiving oxygen at 2 L/min through a nasal cannula.

Approximately one hour after initial stabilization, while under close observation, the patient developed recurrent generalized urticaria, facial angioedema, and chest tightness. There was no stridor or wheezing. Her BP remained stable at 110/80 mmHg, her HR was 100 beats per minute, and her oxygen saturation remained 97% while she was receiving oxygen at 2 L/min via nasal cannula. Given the recurrence of symptoms after complete initial resolution and in the absence of further exposure to the suspected trigger, a biphasic reaction was suspected. Because there was no objective respiratory compromise and the patient remained hemodynamically stable, the treating emergency team elected not to administer an additional dose of epinephrine. She was treated with intravenous hydrocortisone 100 mg and chlorphenamine 10 mg, after which her symptoms completely resolved within 20 minutes.

The patient's home medications included a combined oral contraceptive pill (ethinylestradiol 30 mcg/drospirenone 3 mg) for the past five years, alongside intermittent use of esomeprazole 20 mg and ibuprofen 400 mg. She had no history of atopy, bronchial asthma, chronic urticaria, or prior drug allergies. There was no family history of atopic or immunological diseases.

After the recurrent symptoms had resolved, she was evaluated by the inpatient allergy and immunology service. She was conscious, alert, and oriented. Vital signs were stable: BP 110/80 mmHg, HR 70 bpm, temperature 36.9°C, and oxygen saturation 99% on room air. The remainder of the physical examination was unremarkable, except for a mild, non-pruritic, localized 2×2 cm erythematous plaque at the abdominal injection site.

Diagnostic evaluation, including a 12-lead electrocardiogram and chest radiography, was unremarkable. Baseline laboratory findings upon admission are presented in Table 1.

**Table 1:** Laboratory findings at initial presentation to the emergency department following tirzepatide administration.

Parameter	Result	Reference Range
White blood cells	12.86 × 10^9^/L	4-10
Hemoglobin	14.2 g/dL	12-16
Platelets	444 × 10^9^/L	150-400
Urea	4.6 mmol/L	2.5-7.1
Creatinine	59 µmol/L	44-80
Sodium	135 mmol/L	135-145
Potassium	4.4 mmol/L	3.5-5.1
Alanine transaminase	12 U/L	<35
Aspartate aminotransferase	16 U/L	<35
Total cholesterol	6.31 mmol/L	<5.2
Glycated hemoglobin (HbA1c)	5.5%	<5.7%
Thyroid-stimulating hormone	0.478 mIU/L	0.4-4.0

An acute serum tryptase level was not obtained because testing was unavailable, and a subsequent baseline tryptase level was not measured. Validated skin-testing concentrations for tirzepatide have not been established, and no commercially available tirzepatide-specific IgE assay is available; therefore, the underlying mechanism could not be determined.

Given that serum tryptase and confirmatory allergy testing were unavailable, anaphylaxis was diagnosed clinically according to World Allergy Organization criteria. Tirzepatide was considered the most likely trigger because of the close temporal relationship between administration and symptom onset, the absence of another apparent exposure, and the clinical improvement following treatment and avoidance of further doses [4].

The patient was transferred to the ward for extended monitoring. She was maintained on scheduled intravenous hydrocortisone 100 mg every eight hours and oral cetirizine 10 mg once daily. Given the severity of the initial reaction, the requirement for two doses of intramuscular epinephrine, and the recurrence of symptoms, the patient was observed for 36 hours. She remained clinically stable throughout the remainder of her hospital course and was discharged with a prescription for a 0.3-mg epinephrine autoinjector. The on-call allergy and immunology service provided autoinjector training and an emergency action plan. She was also prescribed oral prednisolone 40 mg once daily for five days, instructed to avoid any further exposure to tirzepatide, and referred for outpatient allergy and immunology follow-up.

## Discussion

The clinical use of dual GIP/GLP-1 receptor agonists, including tirzepatide, has increased substantially, particularly among individuals receiving treatment for type 2 diabetes mellitus or chronic weight management [1,2]. As exposure expands across increasingly diverse patient populations, rare adverse drug reactions that may not have been fully characterized during clinical trials are more likely to be encountered in routine practice [3].

A recent retrospective observational analysis of 43 published adverse-drug-reaction cases associated with tirzepatide found that gastrointestinal and metabolic complications predominated, whereas reports of severe systemic hypersensitivity remained uncommon [3]. The present case is notable for the rapid onset of anaphylaxis following the patient’s first known dose of tirzepatide and for a subsequent recurrence of symptoms suggestive of a possible biphasic reaction. After complete resolution of the initial episode following two doses of intramuscular epinephrine, the patient remained asymptomatic for approximately one hour before developing recurrent generalized urticaria, facial angioedema, and chest tightness without further exposure to the suspected trigger. This recurrence following a symptom-free interval was considered suggestive of a possible biphasic anaphylactic reaction. Biphasic anaphylaxis is characterized by the recurrence of symptoms after complete resolution of the initial episode, without re-exposure to the causative agent, and may occur several hours after the initial presentation [5]. In view of the severity of the initial reaction, the requirement for repeated epinephrine administration, and the recurrence of symptoms, the clinical team elected to continue observation for 36 hours.

The immunological mechanism underlying tirzepatide-associated hypersensitivity has not been established [3]. Immediate hypersensitivity reactions may occur through either IgE-mediated or non-IgE-mediated pathways [4,5]. An IgE-mediated mechanism would require prior sensitization to an immunologically related antigen, although the patient reported no prior exposure to tirzepatide or any other GLP-1 receptor agonist. Alternatively, non-IgE-mediated mast cell activation or complement activation may produce a similar clinical presentation [4,5]. However, no specific mechanism can be inferred from the timing of the reaction alone.

An acute serum tryptase level was not obtained because testing was unavailable at the treatment facility, and a subsequent baseline level was not measured. Furthermore, validated skin-testing concentrations for tirzepatide have not been established, and no commercially available validated tirzepatide-specific IgE assay is available. Consequently, the underlying mechanism could not be confirmed. The diagnosis of anaphylaxis was based on the acute onset of cutaneous, respiratory, and cardiovascular involvement. Tirzepatide was considered the most likely trigger because of the close temporal relationship between administration and symptom onset, the absence of another identifiable exposure, and the improvement following treatment and avoidance of further doses. Nevertheless, in the absence of confirmatory investigations, definitive causality cannot be established.

Most hypersensitivity reactions reported in tirzepatide trials and published safety reviews have been mild and predominantly cutaneous, including urticaria and injection-site reactions [1-3,8]. Severe systemic reactions, including anaphylaxis, appear to be rare, and their true frequency remains uncertain [3,8-10]. Previously published reports include a biphasic anaphylactic reaction and an immediate systemic allergic reaction following tirzepatide administration [9,10]. The present case illustrates that a severe reaction may occur after the first known dose and in a patient without a previous history of atopy, asthma, chronic urticaria, or drug allergy. Therefore, the absence of recognized allergic risk factors should not be interpreted as eliminating the possibility of an immediate hypersensitivity reaction.

This report has several limitations. An acute serum tryptase level was unavailable, a subsequent baseline level was not obtained, standardized allergy testing was not performed, and no post-discharge follow-up information was available. During the recurrent episode, the patient experienced chest tightness but had no documented wheezing, stridor, hemodynamic instability, or deterioration in oxygen saturation. Consequently, although a biphasic reaction was suspected, the recurrent episode cannot be definitively classified as biphasic anaphylaxis. Rechallenge with tirzepatide was not undertaken because of the severity of the initial reaction and would not have been clinically appropriate. The absence of follow-up also prevented assessment of subsequent allergic events and the outcome of the planned outpatient allergy evaluation. As post-marketing safety data continue to accumulate, careful documentation and pharmacovigilance reporting of suspected severe hypersensitivity reactions will be important for defining their frequency, clinical spectrum, and potential mechanisms [3,8].

## Conclusions

Tirzepatide-associated anaphylaxis is uncommon but potentially life-threatening. This case illustrates that a severe immediate hypersensitivity reaction may occur after the first known dose, even in a patient without a history of allergic disease, and may be followed by recurrent symptoms suggestive of a biphasic reaction. Anaphylaxis remains a clinical diagnosis, and the absence of confirmatory biomarkers or validated allergy testing should not delay treatment. Prompt intramuscular epinephrine remains the cornerstone of management when anaphylaxis is suspected. Continued pharmacovigilance and detailed case reporting are needed to better define the frequency, clinical spectrum, and underlying mechanisms of tirzepatide-associated hypersensitivity.
